# Case Report: Individualization of Intensive Transactional Analysis Psychotherapy on the Basis of Ego Strength

**DOI:** 10.3389/fpsyg.2021.618762

**Published:** 2021-06-09

**Authors:** Irene Messina, Francesco Scottà, Arianna Marchi, Enrico Benelli, Alessandro Grecucci, Marco Sambin

**Affiliations:** ^1^Universitas Mercatorum, Rome, Italy; ^2^Centro Psicologia Dinamica, Padua, Italy; ^3^Department of Philosophy, Sociology, Education and Applied Psychology, University of Padua, Padua, Italy; ^4^Department of Psychology and Cognitive Sciences, University of Trento, Rovereto, Italy

**Keywords:** ITAP, dynamic psychotherapy, single-case, outcome, brief dynamic therapy, process, ego strength

## Abstract

In intensive transactional analysis psychotherapy (ITAP), intensity is obtained with both technical expedients and the relational manner with the patient. In ITAP, the therapist modulates pressure and support commensurately to the patients' ego strength. In the present article, we contrast two clinical cases of young adults in which ego strength produced different therapy outcomes and processes. We present excerpts of the psychotherapy process that illustrates technical aspects of ITAP as well as the therapist's attitude that we describe as holding. We show quantitative therapy outcomes consisting of effects size values of changes in Clinical Outcome in Routine Evaluation—Outcome Measure scores in baseline, treatment, and follow-up phases and qualitative outcome evaluated with the Change Interview at the end of the therapy. In the patient with high ego strength, we observed a rapid improvement and a complete recovery at the end of the therapy, whereas the results of the patient with low ego strength were less consistent (more fluctuations in Clinical Outcome in Routine Evaluation—Outcome Measure scores including deterioration but good qualitative outcome). We conclude that quantitative and qualitative outcome data, together with process observations, are required to have a complete picture of therapy effectiveness. Moreover, we conclude that qualitative ego strength is not a limitation for the use of expressive therapy such as ITAP, but rather, it is an important variable that should be considered to dose confrontations and support.

## Introduction

Intending to increase intensity in therapeutic intervention, intensive transactional analysis psychotherapy (ITAP) is short-term psychodynamic psychotherapy that integrates transactional analysis (Berne, 1961; Schiff, 1975; Goulding and Goulding, 1979) with brief psychodynamic psychotherapy approaches (Malan, 1976; Davanloo, 1994; Fosha, 2000; Abbass, 2015). In ITAP, intensity is considered to be related to the therapist's activity, which is enhanced with both technical expedients and the relational manner with the patient.

At the technical level, the intrapsychic triangle and the interpersonal triangle are used by ITAP therapists for the optimization of interventions in a psychotherapy session. The intrapsychic triangle guides the therapist in analyzing intrapsychic dynamics among impulse, anxiety, and defense (Menninger, 1958; Malan, 1976; Davanloo, 1994). According to the psychoanalytic tradition, an *impulse* is a manifestation of the Id (Freud, 1923). In the ITAP model, it is broadly defined as any spontaneous manifestations of the functioning of the person, including a person's emotions, needs, creativity, and aspirations. *Anxiety* is a negative emotional activation that emerges in the presence of an obstacle to the satisfaction of impulses. Thus, anxiety is a signal of internal danger for the person (Freud, 1923). Educational and social restrictions are examples of obstacles to the satisfaction of impulses. Against impulse and related anxiety, the person may unconsciously use *defenses* (Frederickson et al., 2018; Grecucci et al., 2020a,b). Several defense mechanisms have been described in psychoanalysis, from forms of avoidance of disturbing thoughts or memories (e.g., denial or suppression) to severe distortions of reality (e.g., projections or delusions) (Vaillant, 1992). In ITAP, the therapist aims for impulse emersion. In pursuing this aim, he/she notes every anxiety manifestation as a signal of a covered impulse, and he/she confronts the patient with defenses that blocks the emersion of the impulse.

The intrapsychic triangle is used jointly with the interpersonal triangle, which guides the therapist in analyzing repetitive relational patterns in the person, exploring such patterns across different relational situations (Menninger, 1958). Here-and-now relational difficulties reported by the patients (*current relationship*) are explored, comparing them with relational experiences with the therapist in psychotherapy sessions (*therapeutic relationship*) and with *past relationships* in which repetitive relational patterns may have been formed as an effect of traumatic experiences. Thus, in the ITAP model, psychic functioning is described as interconnections of impulse, anxiety, and defenses, which have originated in past relationships and which can be enacted in here-and-now relationships (current relationships and/or therapeutic relationship) (Sambin, 2018a).

At the relational level, the therapist modulates the technique based on the level of the patient's anxiety manifestations, *holding* the patient during the exploration of intrapsychic and interpersonal triangles. The concept of *holding* refers to a relational attitude characterized by the full presence of the therapist in the relationship, with a moment-by-moment evaluation of the resources made available by the patient throughout the session (Scottà, 2018). On the basis of available resources, the therapist modulates *pressure*—a very active attitude, which intensifies psychotherapy sessions by moving the attention of the patient through the various vertexes of the ITAP triangles—and *support* (Sambin, 2018b). In other words, the therapist applies pressure and support commensurately according to the ego strength, a psychodynamic concept referring to a set of capacities including individual resilience, identity integration, personal resources, ability to maintain satisfactory interpersonal relationships, and self-esteem (Freud, 1923; Lake, 1985). Thus, ego strength may strongly influence the actual duration and intensity of ITAP, as well as the evolution of the psychotherapy process toward the psychotherapy outcome.

Psychotherapists have long realized that treatment should be tailored to the individuality of the patient. As part of the *what works for whom* approach (Roth and Fonagy, 2006; Norcross and Wampold, 2011), the identification of effective methods of adapting treatment to the individual patient (other than diagnosis) has become an object of investigation in psychotherapy research. Among individual factors, ego strength has been reported previously as being predictive of psychotherapy outcome (Barron, 1953; Conte et al., 1991; Laaksonen et al., 2013; but see also: Getter and Sundland, 1962). Also, variables attributable to ego strength, such as personality impairments in the patient (Hersoug et al., 2013), self-concept, and quality of object relations (Lindfors et al., 2014), have been associated with worse outcomes. With the present article, we contribute to this line of research by contrasting two clinical cases in which ego strength—the main element of calibration of intensity in ITAP—produced different therapy processes and outcomes. We consider that single-case methodology can be particularly suitable for the investigation of individual factors (Messina et al., 2018, 2019). It allows longitudinal evaluations with a large number of observations to look in detail at how change unfolds over time during the therapy of each specific patient. Also, a single-case methodology is compatible with the use of qualitative measures that may be helpful in clarifying the influence of individual and contextual factors. In addition to quantitative and qualitative outcome measures, we also present excerpts of the psychotherapy process that illustrates (a) the impact of the use of ITAP triangles on impulse emersion and (b) therapist's attitude that we describe as holding.

## Method

### Instruments

#### Assessment of Ego Strength

Patients' ego strength was evaluated by the research team using the *structure* axis of the *Operationalized Psychodynamic Diagnosis* system (OPD-2; OPD Task Force, 2008). According to the Structure axis of the OPD-2 system, the psychic structure of the patient (or his/her ego strength) can be classified as well-integrated, moderately integrated, low integrated, or disintegrated, on the basis of the following markers: (a) Cognitive abilities (self-perception and perception of the object); (b) Regulation (self-regulation and regulation of the object relation); (c) Emotional communication (internal communication and communication with the outside world); (d) Attachment (internal objects and external objects).

#### Quantitative Assessment of Psychotherapy Outcome

Psychotherapy outcome was evaluated quantitatively through the Clinical Outcome in Routine Evaluation—Outcome Measure (CORE-OM). The CORE-OM is a widely used scale for the routine evaluation of psychotherapy outcomes (Barkham et al., 2001; Evans et al., 2002). It is composed of 34 items that are scored on a 0–4 scale (from 0 = “*Not at all*” to 4 = “*All or most of the time*”). CORE-OM global scores allowed the classification of the patients on the basis of their distress level: *healthy* (score <0.6), *low-level* (score between 0.6 and 1.0), *mild* (score between 1.0 and 1.5), *moderate* (score between 1.5 and 2.0), *moderately severe* (score between 2.0 and 2.5), or *severe* (score >2.5). Moreover, four subscales allowed the evaluation of four outcome variables: well-being, psychological problems (depression, anxiety, somatic problems, and trauma), functioning (general functioning and functioning in close relationships and social relationships), and risk (risk to self and others). The Italian version of the CORE-OM shows good acceptability, internal consistency, and convergent validity (Palmieri et al., 2009).

#### Qualitative Assessment of Psychotherapy Outcome

Psychotherapy outcome was evaluated qualitatively through The Change Interview, a semi-structured interview that provides qualitative descriptions from patients of perceived change reported at the end of the therapy (Elliott et al., 2001). Patients are asked to identify the most relevant changes they made during the therapy and to evaluate them on a five-point scale: (a) if he/she expected the change (from 1 = *expected change* to 5 = *surprising change*); (b) how likely these changes would have been without therapy (from 1 = *unlikely* to 5 = *likely* without therapy), and (c) how important he/she feels these changes to be (from 1 = *slightly important* to 5 = *extremely important*).

### Participants

#### Patients

Two young adult patients differing in ego strength as evaluated with the OPD-2 were selected from a larger clinical study testing ITAP efficacy. Diagnostic and Statistical Manual of Mental Disorders, 5th Edition (DSM-5) diagnosis was provided for each patient, and they were asked about the goals of their therapy before starting the treatment. For both patients, this was the first experience of psychotherapy, and they were not treated pharmacologically.

#### Maria

Maria was a patient with a *well-integrated* structure according to the OPD-2 diagnosis. She was a 25-year-old female student. In her therapy, she focused mainly on her relational difficulties. She reported having difficulties in regulating her emotions with others. On the one hand, she suffered because sometimes she was aggressive with others, and then, she felt guilty as a consequence of this aggressiveness. On the other hand, she perceived not being free to express herself with her family, and she wanted to feel free to make her own decisions. She also suffered from anxiety and loss of concentration. In addition to these emotional difficulties, she wanted to cope with the loss of her dog (which was living with her ex-partner). With regard to the diagnosis, she saturated the DSM-5 criteria for *dysthymic disorder* and *generalized anxiety disorder* (American Psychiatric Association, 2013). Maria's CORE-OM scores at baseline were in the clinical range, except for the functioning score that was in the normal range (see Table 1 for scores). The total CORE-OM score was situated in the *mild* range of distress.

**Table 1:** CORE-OM scores at baseline and treatment + follow-up.

**CORE-OM scores**										
	**Patients**	**Baseline**		**Treatment**		**Follow-up**		**Baseline vs. Treatment** ***Hedge's g***	**Baseline vs. Follow-up** ***Hedge's g***	**Treatment vs. Follow-up** ***Hedge's g***
		**Mean**	**SD**	**Mean**	**SD**	**Mean**	**SD**			
**Well-being** Clinical threshold: F 1.84 M 1.40	Maria	1.88	0.60	0.73	0.41	0.50	0.43	2.44***	2.64***	0.53
	Fabio	1.44	0.13	1.70	0.63	1.17	0.29	−0.43	1.08**	0.84*
**Psychological Problems** Clinical threshold: F 1.44 M 1.20	Maria	2.40	0.34	0.91	0.50	1.11	0.09	2.99***	4.03***	−0.41
	Fabio	1.29	0.16	1.61	0.55	1.31	0.29	−058*	−0.07	0.54*
**Functioning** Clinical threshold: F 1.31 M 1.29	Maria	1.19	0.36	0.74	0.33	0.83	0.17	1.28**	1.01**	−0.27
	Fabio	1.15	0.08	1.56	0.42	1.39	0.17	−1.01**	−1.62***	0.41
**Risk** Clinical threshold: F 0.22 M 0.25	Maria	0.33	0.31	0.13	0.24	0.22	0.38	0.75*	0.27	−0.33
	Fabio	0.08	0.16	0.15	0.24	0.28	0.09	−0.29	−1.23**	−0.54*
**Total Score** Clinical threshold: F 1.20 M 1.09	Maria	1.45	0.29	0.59	0.20	0.67	0.06	3.76***	2.88***	−0.40
	Fabio	0.99	0.08	1.26	0.41	1.03	0.08	−0.68*	−0.42	0.57*

Interpretation of Effect Size (ES) value: >0.02 = small effect; >0.50 = medium effect (*); >0.80 large effect (**); >1.30 very large effect (***).

#### Fabio

Fabio was a patient with a *low-integrated* psychic structure, according to the OPD-2. He was a 24-year-old student. In his therapy, Fabio's main goal was to cope with severe anxious symptomatology that included social anxiety, claustrophobia, and panic attacks characterized by tunnel vision and temporary loss of reality perception. He reported having very low functioning in social relationships, with feelings of discomfort and freezing in social situations, conditions that fomented strong internal judgment and feelings of guilt that were the object of disturbing and continuous rumination. In the face of these difficulties, he wanted to become more spontaneous in social interactions. Fabio saturated the DSM-5 criteria for *panic disorder* in axis I and *schizoid personality disorder* in axis II. Although Fabio reported severe symptomatology and the therapists evaluated his personality as being low structured, Fabio's total scores compared with Italian normative data were within the nonclinical range at the beginning of the therapy (see Table 1).

#### Therapists

The same 32-year-old male therapist treated the patients. He is one of the founders of the ITAP approach, an expert in transactional analysis and brief dynamic therapy. He had a formal 4-year clinical training as a psychotherapist and had 3 years of experience in doing psychotherapy after training. The therapist discussed each clinical case in regular group supervision with the research team.

#### Research Team

In addition to the therapist, the research team was composed of three experienced researchers with both scientific (doctor of philosophy) and clinical training as psychotherapists and three advanced students. Two of the experienced researchers also had specific training as psychotherapy supervisors. The students participated in research team/clinical supervision groups, and they were also involved in data collection and analyses.

### Procedures

#### Recruitment and Ethical Issues

Patients were recruited from a waiting list of students who had psychological or relational difficulties and were voluntarily referred to therapy as part of a larger clinical study. The patients were voluntary students attending the same university as the research team, but they had no direct connection with the research team. The Ethical Committee of the University of Padua approved the research protocol. Before entering treatment, all patients received detailed descriptions of the research protocol, and they were informed that they were free to leave the research protocol at any moment without consequences for the continuation of their therapy. In the informed consent, a specific section for the use of video-recorded sessions was included, and it was specified that patients would not be identifiable on the basis of the material presented in scientific publications.

#### Data Collection

For the evaluation of psychotherapy outcome time series, longitudinal data were collected in three different phases: (a) *Baseline* included 5 weekly evaluations in 5 consecutive weeks before the beginning of the therapy (with the last evaluation immediately before the first session); (b) *treatment* included weekly evaluations realized immediately before each session (with the first evaluation immediately before the second session); (c) *follow-up* included evaluations realized at 1, 3, and 6 months after the end of the therapy. For each assessment, patients filled out the CORE-OM in the clinical psychology laboratory and in the presence of an external research assistant. During the first follow-up, a researcher carried out the *Change Interview* to collect qualitative data concerning patients' subjective perception of changes. Patients were informed that the therapist had no access to any research data provided.

#### Therapy

The treatment followed the procedures described in the ITAP manual (Sambin and Scottà, 2018). Sixteen sessions of ITAP therapy were planned as part of the research protocol. Maria had the planned number of sessions, whereas four additional sessions were provided to Fabio due to the clinical evolution throughout his therapy (see Results). The sessions were 50 min, with weekly frequency, with a total time of 4 months of treatment for Maria and 5 months for Fabio. The therapy was provided free of charge, and the patients were informed that they could withdraw from the study at any point, without any negative impact on their therapy.

## Process Data

### Impulse Emersion

Here, we present two excerpts of the therapy of Maria and Fabio to illustrate how ITAP works. Each excerpt is introduced by a brief description of the context of what was occurring in the session and is followed by a brief conceptualization of the event in line with the ITAP model. The excerpts are verbatim transcripts with ellipses to show where words were deleted to shorten the presentation, and minimal encouragers (e.g., “Mm-hmm”) were dropped unless they had specific communication value. In brackets, we reported the position in the intrapsychic triangle (A = Anxiety, D = Defenses, or I = Impulse) and interpersonal triangle (P = Past, C = Current, or T = here-and-now in psychotherapy). Regarding the therapist's interventions, the positions to which the therapist moves are preceded by the symbol → (e.g., if the therapist explores or emphasizes an I/C, we use the symbol “ → I/C”). In few cases, interventions escape from triangle classifications. Thus, we provided few additional categories. “Aw” refers to therapists' interventions aimed at stimulating aspects of awareness in the patients (→Aw) and the patient's responses indicating the acquisition of aspects of awareness (Aw); “E” refers to empathic interventions; “Al” refers to therapists' interventions aimed at the alliance. According to the consensual qualitative research method (Hill et al., 2005), research team members discussed to reach a consensus for the assignment of a category to each intervention.

#### Excerpt From Maria

This excerpt is taken from the eighth session. In this excerpt, the patient talks about an episode in her current life (C): she recently encountered a dog similar to the one she had to leave with her ex-boyfriend and became sad. The therapist aims to bring out the impulses activated in this episode by comparing the patient's defensive modes (D) in the here-and-now of therapy (T) to the emotions related to the loss of the dog (I). The patient is able to achieve a greater awareness regarding her tendency not to face conflict situations by giving in to the will of others (such as leaving her beloved dog to her ex-boyfriend).

T: *What did you feel in that moment?* [→ I/C]
M: *I don't know how to express it, I mean it was really a strange thing…a lump in my throat…* [A/C]
T: *How was this lump in the throat for you?* [→ A/C]
M: *Nice and bad…Nice because it was nice, I mean, ah, it took my back to another world in a moment … And bad because once it's finished you think in any case “Who knows what will happen to my dog…”* [D/C]
T: *As if you'd realized that that scene isn't there anymore?… How did you feel in that moment?* [→ I/C]
M: *Ah, the darkness takes you* [I/C]
T: *You mean, this scene was sad?* [→ I/C]
M: *Yes, yes, also. Even now that I'm talking about I feel the darkness returning, yes …* [I/T]
T: *Yes, but you're laughing a lot* [→ D/T]
M: *Ah I know, well, unfortunately it's a bad habit of mine, laughing* [D/T]
T: *No, I have the impression that there's a part of you that's sad, and another part that says “No, no, come on, everything is ok, laugh about it”* [→ D/T]. *But a part of you is sad* [→ I/T]
M: *Ah yes, I can't get rid of it, I mean I can't get rid of a piece of my life, get rid of some memories. No?* [Aw/C]
T: *But the memories are sad…* [→ I/T]
M: *Ah yes, but, but you've got to deal with them* [D/T]
T: *And how do you deal with them? IF you deal with them by laughing and then they come back* [→ D/T].
M: *Ah… I don't know another way… I mean the time, I've always said “In time things will pass”, sure enough time has passed a lot, ah, I mean that …* [D/T]
T: *Yes of course. I was concerned about the part, actually, that is worried …that then becomes darkness …* [→ A/T]
M: *I hope not. I mean, I hope that… this doesn't happen, I hope so. I mean every day of my life … to do things that make me so satisfied that I don't think of anything else, no?* [D/T]
T:…*I have the impression that not thinking about it creates a, sort of, barrier for a bit* [→ D]*, then something bigger comes along …* [→ I/T]
M: *Yes, yes, I've thought about this …* [Aw/T]
T: …*the barrier collapses and everything that wasn't there before comes along …* [→ D/T]
M: *Yes, yes, it's true …* [Aw/T]
T: *And I'm worried about this, because the barrier of doing stuff so as not to feel what there is over here* [mimes a barrier with the hand], *it holds up a little, a little and then by dint of doing this you get all of the manifestations* [he points to her arm, on which the patient had a cutaneous eruption], *and then, as is natural, it collapses. And when it collapses it's a month and a half, two, of darkness*. [→ D/T]
…
T: *What are you in contact with?* [→ I/T]
M: *I don't know what, I don't know what this thing inside of me is, I'm trying to bring out something that's inside me that I don't know*. [D/T]
T: *On a cognitive level yes, I have the impression that you don't know. On an emotional level how are you, when you think of these things? Actually, when do you feel this thing?…Let's try to remain there, to listen to what's there, behind that barrier that I was talking about before …*[→ I/T]
M: [Silence] *I don't know, because I was different, I was like other people, as if I was talking about other people, a lot of things have changed, so I can no longer reflect myself in what I was. I have really changed personality so I can't remember anything at all*. [D/T]
T: *What is it that's coming back then…?* [→ I/T]
M: *The sensations come back, of nostalgia. I mean, it's the emotions that come back up, nostalgia, anger, it's not the memory… The emotions, I mean the impotence*.[I/T]
T: *What are you feeling now, the impotence?*[→ I/T]
M: *Yes*. [I/T]
T: *Is that what you couldn't get a handle on?* [→ I/C]
M: *Yes, exactly maybe the impotence of not having - I as I do generally - I mean that I let things go rather than assert myself on things, I don't assert myself on things … Because I don't want to get to a discussion…* [I/C]*Like when S. says to me “Oh, the dog's staying with me, because I can provide it with more things”…* [Aw/C]
T: *And you want that dog?*[→ I/C]
M: …*Yes, I want it* [I/C]*, but I can't, I made this choice* [D/C]…
T: *Yes, yes… on a cognitive level it seems very clear: “I chose this”*.
M: *Ok, on an emotional level. Ah no, because clearly it wasn't good for me*.
T: *And that thing there comes and returns, cyclically* [→ I/C].
…
M: [the patient talks about how recently she is feeling the necessity to assert herself in various contexts] …*now I am starting to reason in a much more selfish way* [Aw/C]
T: *Ah there you are, if you could think in a selfish way when you're with L?*[→ I/C]
M: *Ah I'd like to give him a slap it's different* [I/C]…*but there as well, what's the point of it…?*[D/C]
T: *There's the sense of listening to what you feel. That's it, what you feel. As a fantasy, if you could what would you do to this L?*[→ I/C]
M: *Ah, I'd gladly give him a few slaps* [I/C]
…[the patient stimulated by the therapist expresses her anger through the use of fantasies]
T: *You knocked it down, and you knocked it down, and you knocked it down… and now, luckily, it's coming up, it's coming up, it's coming up…*
M: *I had enough…That strength I… I've always had it inside*.[Aw/P]
T: *Very good, meanwhile let's try to understand what there is underneath it, that reservoir that stayed there and let's start to knock it out, and to process it…Have you seen that we've caught our fish: you are very angry with him still … Can you accept it?*
M: *Yes!*[Aw/T]

In the therapy extract, we can observe some elements that indicate a good level of ego strength in the case of Maria. First of all, it can be observed that anxiety is present at manageable levels and is mainly discharged at the level of the striated muscles (e.g., a lump in the throat). As typically happens with this type of manifestation of anxiety, the defenses are of an evolved type and concern the recognition of emotional aspects on a cognitive level with an avoidance of the actual emotion (the patient smiles while telling a sad episode so as not to come into contact completely with the sadness) or the repression (“I have really changed personality so I can't remember anything at all.”). Finally, we see that Maria manages to understand the therapist's interventions without getting too anxious and easily acquires aspects of awareness.

#### Example From Fabio

This excerpt is taken from the third session. The patient starts with an episode from his own past (P) in which he recounts a situation where he had been very frightened, and his fear had not been accepted sufficiently. The patient easily links the terror he felt in P with the terror he currently feels during his anxiety crisis (C). The therapist encourages Fabio to focus on his emotions in recalling that episode in the here-and-now of the session (T), helping the patient to recognize some defensive tendencies and to get in touch with his own impulses of sadness (I).

F: *I was on my way home and there was someone there, I met someone who was like, “Hey there, who are you?” Ah I got scared for a moment no, pretty scared for a child…Then this person I saw - then maybe I imagined - that he was following me from behind, so I had a moment and started running*. [I/P]
T: How scary![→ I/P]
F: *Oh yes quite so… there was nothing there, it was in the middle of nowhere, to get home, that is there are only fields and so I was alone there… And then I was like this* [indicates a child's height]*, the other guy was like this* [indicates an adult's height]… [I/P]
*T: And so you were very scared* [→ I/P]
*F: Yes, exactly* [I]
*T: And so every time you went that way, you relived that fear?* [→ I/P]
*F: Yes pretty much* [I]
*T: Have you ever had a chance to talk to anyone about that moment?… was your fear somehow acknowledged?* [→ I/P]
*F: Yes, it was acknowledged, but I couldn't find a solution. So…*.
*T: Ok*.
F: *My dad told me, he said, “Look, don't worry about it…” I mean, a reassurance that's a little too rational, that's all*.
T: *He didn't listen to you. (he hugs his belly)*
*F: Exactly*.
T: *That kid was still worried* [I/T].
F: *Yes, terrified* [I/T]
T: *Terrified. By others?*[→ I/C]
F: *Well, in this case yes, well now that you mention it this terror maybe with the panic attack comes back a bit when… for example in a deserted street like I told you…* [I/C].
T: *Is this memory useful to you? I mean, this connection that you're making?* [→ Aw]
F: …* Well, it's useful because I see a similarity between the terror felt in both cases*. [I]
T: *The terror of the child being left alone with maybe someone following him in the fields… …* [I/P]
F: *Yes. (I/P)*
T: *And terror of the adult who, on the other hand, how can I put it, connects, links up…* …[→ Aw of the link between I/P and I/C]
F: *Yes*.
T: *with the terror of the child*. [Aw of the link between I/P and I/C]
*F: Because it is the same terror in those moments when the panic attack… in fact I feel like a child… I feel in the middle of the fields, lost, small… helpless even*. [Aw]
*T: Small, helpless, scared*.
*F: Scared*.
…
T: *Where are we now, out of these things?* [→ I/T]
F: *in this moment, sadness* [I/T]
T: …*as if we had also evoked the sadness of when you were a child… That child was feeling so many things* [→ I/T]
F: *Yes, quite… I've always made things complicated*. [D/T]
T: *You're judging yourself* [→ D/T]
F: *Yeah, my parents told me I was complicating things* [D/P]. *And now I just remembered that around elementary school - these episodes are all around elementary school - I had to go…* [D/T]
T: *Can I stop you for a moment?* [→ Al]
F: *Yes*. [Al]
T: *I think it's useful to stop, otherwise we'll move on to more cognitive aspects…* [→ Al]. *Remember that it' s all right, it's all right*. [→ E]… *But it's like we're jumping a little bit away from these emotions* [→ D/T]
F: *Ah ok* [Al]
T: *It's not a judgment, no one is to blame, it's okay*. [→ E]. *But I think it's useful for you to stay on these emotional issues that have emerged very clearly and very strongly* [→ Al]*, otherwise there's a chance we'll do it the way we did it* [moves his hand, as if to move, to pass over] [→ D/T]
F: *Oh, okay, I get it, yeah, you mean, just distance yourself right away…* [Aw/T]
T: *Distance yourself immediately. Instead we found out that that child was angry, scared, feeling helpless* [→ I/P]… *Now you're feeling these emotions here* [→ I/T]
F: *Ah* [sigh] [A/T]
T: *Ah* [sigh] [E/T]
F: *Ah, it's not simple…*[A/T]
T: *It's not simple* [E/T]. *As far as I can stand them, how can I say this, I'm there, I'm with them, it's a way to be with that child too…we here maybe have the chance to be with that child. If*
*not, we'll leave him alone one more time*. [→ Al].
F: *Ah ah, ok. Yes that's ok*. [Al]
T: *Mm? Do you have it, are you seeing it?*
F: *Yes*.
T: *And what's it like?*
F: *I don't know, I'm picturing him locked in a corner with some bars… crying*. [I/T]
T: *Ah, ok. Mm. And as you see him, what can you do? Now, that you're older*. [→ I/T]
… [the therapist explores a possible Impulse]
F: *Well, I'd give him a hand and caress him, let's say…* [I/T]
T: *Does he feel it?* [→ I/T]
F: *Yes*. [I/T]
T: *And how is he?* [→ I/T]
F: *Ah, warmer, more relaxed* [I/T]
T: *Listen to yourself for a second. Don't use words, there's no need to explain* [→ I].
F: *Ok*. [Silenzio] [I]
T: *Listen to yourself. How's your breathing, how's your body, how is this sensation of warmth?* [→ I]
F: *Calmer, more relaxed. With fewer things running through my mind* [I/T]
T: *Calmer, more relaxed. The warmth calms*. [→ I/T]
F: *Yes. Ah yes, the thoughts as well*. [Connects I and D]
T: *It calms your thoughts as well*.
F: *Yes*.
T: *So even the thought of that child with the hand calms you down*.
F: *Ah yes. Pretty much, yes. But my ears are ringing* [A/T]
T: *Yeah. Okay. All right. It's okay, it's okay. We're working on some important stuff…* [E/T]
F: *Ah ok* [E]
T: *So there is a realignment of your structure right now* [→ Aw]. *Do you follow me?* [→ Al]
F: *Ah ok. Yes yes yes yes yes. Yes yes yes yes …*.[A/T] [the patient motions, indicating that he can hear the ringing in his ears…]
T: *Have your ears started ringing?* [→ A/T]
F: *My ears have started ringing* [A/T]
T: *Was there also a feeling of movement a little bit inside? I mean 'oops'!* [→ A/T]
F: *Yes, exactly yes* [Aw].
T: …*You' re becoming aware of yourself in a different way from the way you were before, you're in contact with a part of yourself, emotionally and physically, as you weren't before…*[→ Aw].

In this second therapy extract, we can observe certain elements that are indicative of a low level of ego strength in Fabio's case. It can be noted that the anxious manifestations also involve cognitive-perceptive aspects (ringing in the ears), as well as those concerning the striated muscles (being stuck to the chair) (Abbass, 2015). To deal with these high levels of anxiety, the therapist uses many interventions of empathic validation and alliance verification, an attitude that highlights the holding attitude. Despite the low level of ego strength, the therapist, through his constant holding, allows Fabio to contact different aspects of impulse and to acquire some elements of awareness.

## Outcome Data

### Quantitative Outcome

To quantify change, we calculated Hedge's g value for a corrected effect size (ES) of change in CORE-OM scores (global score, well-being, psychological problems, functioning, and risk) from baseline *vs*. treatment phases, baseline *vs*. follow-up phases, and treatment *vs*. follow-up phases (Rosenthal, 1994). The calculation of Hedge's g is based on the subtraction of the mean of one group from the other (M1–M2) and the division of the result by pooled the standard deviation. Both comparisons, “baseline vs. treatment” and “baseline vs. follow-up,” provided data concerning pre- vs. post-therapy; however, the former was influenced by fluctuations in the score during the therapy, whereas the latter was not. The additional “treatment vs. follow-up” comparison was useful in evaluating the maintenance of improvements obtained in the treatment phase.

#### High-Functioning Patient

**Figure 1:**
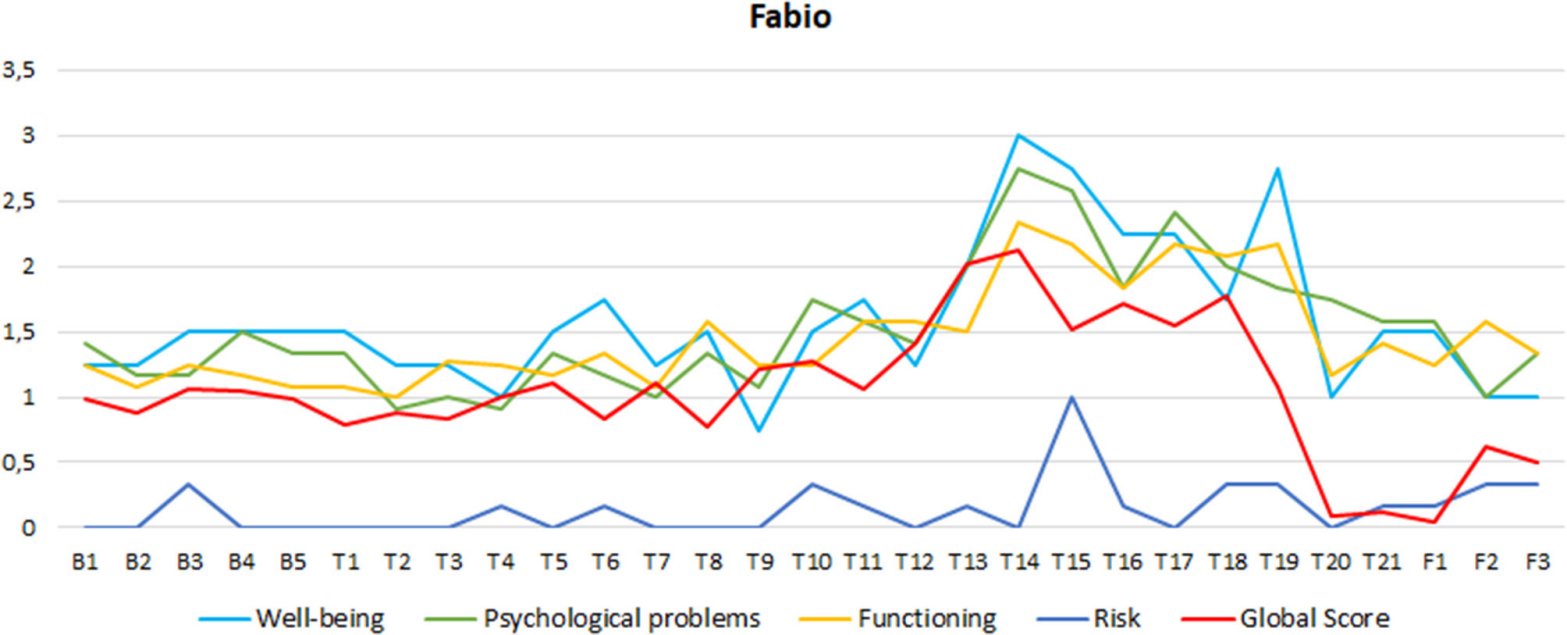
Fabio's CORE-OM subscales scores in Baseline, (B) Treatment (T) and Follow-up (F) evaluations.

Maria's CORE-OM scores at baseline were in the clinical range, except for the functioning score that was in the normal range (see Table 1 for scores). The total CORE-OM score was situated in the *mild* range of distress. As shown in Figure 1, a rapid improvement was observed in Maria's scores during the early sessions, with scores that decreased from the clinical to the nonclinical range for all CORE-OM subscales and with a global decrease from the *mild* range to the *healthy* range of distress. Thus, CORE-OM scores show a complete recovery for Maria.

This description was confirmed in statistical analysis. In “baseline *vs*. treatment” comparisons, we found very large ES in CORE-OM total scores (ES = 3.76), as well as in subscales well-being (ES = 2.44) and psychological problems (ES = 2.99). A large ES was found for the functioning subscale (ES = 1.28), and a medium ES was also observed for the risk subscale (ES = 0.75).

Similarly, in “baseline vs. follow-up” comparisons, very large ESs were observed in CORE-OM total scores (ES = 2.88), in the well-being (ES = 4.03) and psychological problems subscales (ES = 1.01), and a large ES was observed in the functioning subscales (ES = 1.01). Only a small ES was found in the risk subscale (ES = 0.75) for the “baseline *vs*. follow-up” comparison.

The described improvements were maintained in follow-up evaluations, except for the risk subscale score that increased slightly in the last follow-up (6 months), influencing the global score of distress that moved from the *healthy* to the *low-level* range of distress in the follow-up phase (in the nonclinical range nonetheless). In line with this description, non-relevant changes were observed in the “treatment vs. follow-up” comparisons, indicating the maintenance of achieved CORE-OM scores.

#### Low-Functioning Patient

**Figure 2:**
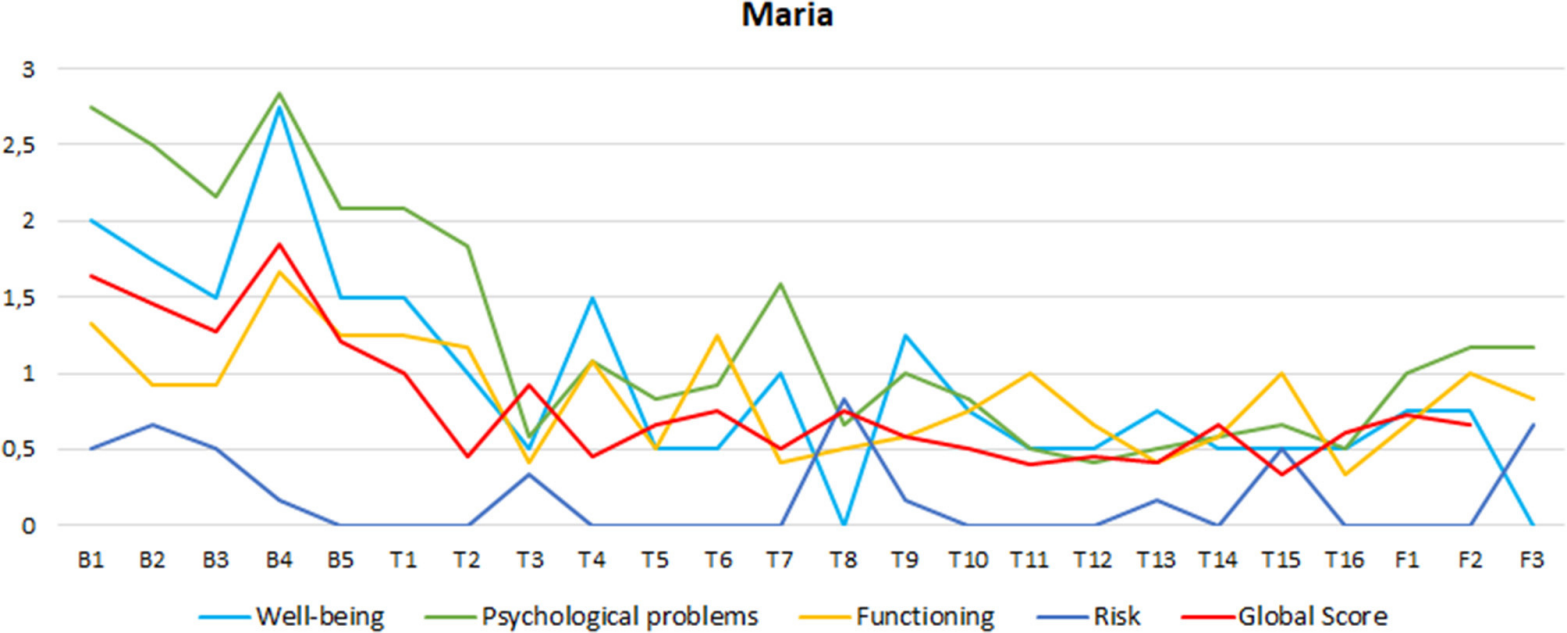
Maria's CORE-OM subscales scores in Baseline, (B) Treatment (T) and Follow-up (F) evaluations.

Although Fabio reported severe symptomatology and the therapists evaluated his personality as being low structured, Fabio's total scores compared with Italian normative data were within the nonclinical range at the beginning of the therapy. As shown in Figure 2, in this therapy, we can observe a progressive deterioration of the patient's CORE-OM score starting from the 11th session, with scores that increase from the non-clinical to the clinical range for almost all CORE-OM subscales, and with a global increase from the *low-level* to the *mild* range of distress. A partial recovery of previous scores was achieved after the 18th session, but it remained in the *mild* range of distress.

The peculiar evolution of this case was also reflected in the statistical evaluations (see Table 1). In “baseline vs. treatment” comparisons, we found a medium effect size indicating the deterioration of the CORE-OM global score (ES = −0.68) and the psychological problems subscale (ES = −0.58). A large ES of deterioration was observed for the functioning subscale (ES = 1.01), whereas non-relevant changes were observed for the well-being and risk subscales.

In “baseline vs. follow-up” comparisons—which is less influenced by fluctuations in the score during the therapy—a large ES indicating improvement was observed for the well-being subscale (ES = 1.08). However, very large and large deteriorations in ES were observed, respectively, for the functioning (ES = −1.62) and risk subscales (ES = 1.23), whereas non-relevant changes were observed in the psychological problems subscale and in the CORE-OM global score.

The partial recovery achieved after the 18th session was maintained in follow-up evaluations. This recovery can be statistically observed in the “treatment *vs*. follow-up” comparisons where a medium ES was obtained for the CORE-OM global score (ES = 0.57), the well-being subscale (ES = 0.84), and the psychological problems subscale (ES = 0.54), whereas a medium ES of deterioration was maintained for the risk subscale (ES = −0.54), and non-relevant changes were observed for the functioning subscale.

### Qualitative Outcome

Although quantitative data indicated a positive outcome for Maria and a negative outcome for Fabio, the qualitative evaluation of the psychotherapy outcome realized using the Change Interview method accounts for a very positive outcome for both patients. They reported several changes classified as very important and extremely important, and they considered many such changes as being unlikely without the therapy. Interestingly, most of the reported changes are in line with the declared aims of ITAP. They concern interpersonal relationships (analyzed with the interpersonal triangle), emotion regulation (analyzed with the intra-psychic triangle), and the improvement of self-representations achieved through contact with self-relevant impulses. Detailed results of the Change Interview are reported in Tables 2, 3.

**Table 2:** Summary change interview of Maria.

**Change**	**How expected the change was**	**How likely/unlikely the change would have been without therapy**	**Importance of change**
*Management of my relationships: before therapy I felt anxious, my relationships were heavy and now they are lighter because now I am more focused on what counts for me*.	4 somewhat surprised	1 unlikely	5 extremely
*I feel calmer when I cope with things*.	1 expected	4 somewhat likely	4 very
*I saw everything as white or black, whereas now I see shades of gray*	5 surprised	1 unlikely	4 very
*I feel good about my body (weight loss)*	5 surprised	5 slikely	5 extremely
*I take care of myself, I take time to relax*	2 somewhat expected	4 somewhat likely	4 very
*Now I feel that I am a valuable person*	1 expected	5 likely	5 extremely
*I can think about myself* [and not only about others]	5 surprised	3 neither	5 extremely
*I am enjoying the fruit of my work, for example at university*	5 surprised	5 likely	5 extremely
*I feel strong, I feel I have power in my hands*	1 expected	1 unlikely	5 extremely
*I accepted the separation from my dog*	1 expected	3 neither	4 very
*I am still harsh with my friends; I have not modified this and in fact I still easily get angry with them I am often on a war footing. However, I have more instruments to manage it*.	1 expected	1 unlikely	4 very

**Table 3:** Summary change interview of Fabio.

**Change**	**How much expected the change was**	**How likely the change would have been without therapy**	**Importance of change**
*I am more spontaneous in relationships with others*.	2 somewhat expected	1 unlikely	4 very
*I don't need to control everything anymore*.	4 somewhat surprised	1 unlikely	4 very
*I am less scared of meeting others outside of my expectations*.	5 surprised	4 somewhat likely	4 very
*I express aspects of my personality that before I used to suppress*.	5 surprised	1 unlikely	3 neither
*Now I deal with the “sergeant”* [Critical Parent or Super-Ego] *and I don't feel him as a superior, now he is my ally*.	5 surprised	1 unlikely	5 extremely
*If I feel frustrated, I try to do better without giving up or criticizing myself*.	1 expected	4 somewhat likely	5 extremely
*I have reduced my armor, I don't expect others to judge me anymore*.	1 expected	1 unlikely	4 very
*I am able to accept my fragility and my limits and to change something instead of criticizing myself*.	1 expected	5 likely	4 very

## Discussion

In the present study, we contrasted two clinical cases of patients with different levels of ego strength (or different levels of psychic structure integration) treated with ITAP, a new psychotherapy approach that aims toward the intensification of therapist intervention through the integration between transactional analysis and brief psychodynamic approaches. Following the *what works for whom* approach, our final aim was to reflect on the possibility that intensive interventions may be differently efficacious in helping patients with different levels of psychic structure integration.

If we consider ITAP outcomes evaluated using quantitative measure, CORE-OM data account for clearly different outcomes in the clinical cases analyzed in the present study. Maria—the patient with a well-integrated psychic structure—obtained a complete recovery, with a rapid improvement in early sessions and the maintenance of these results in follow-up evaluations. This pattern of change corresponds to a typical trajectory of change previously described in the literature (Duckworth et al., 2010; Vittengl et al., 2016). Moreover, these data are consistent with extremely and very important changes associated with the therapy as reported by Maria in qualitative evaluation, as obtained through the Change Interview. Thus, the efficacy of ITAP seems incontrovertible in the case of Maria.

Fabio, the patient with a low-integrated psychic structure, showed more fluctuations in CORE-OM scores during the therapy, and deterioration or non-relevant changes in outcome scores were observed in the “baseline *vs*. treatment” or “baseline *vs*. follow-up” comparisons. At first sight, these results support the hypothesis that ITAP may be more effective for patients with high ego strength compared with patients with more impaired psychic structure. This conclusion would be in line with previous studies showing that psychotherapy outcome is influenced by patients' ego strength (Barron, 1953; Conte et al., 1991; Laaksonen et al., 2013). However, an in-depth reflection is required to define a more realistic picture of Fabio's case. First, studies concerning the psychometric characteristics of CORE-OM have largely demonstrated that initial levels of distress are predictive of subsequent improvement after therapy (CORE Partnership, 2007). Namely, the chance of improvement is negligible for patients with CORE-OM global scores classified as healthy or low level (they cannot recover because they are already “healthy”), whereas it is more likely for patients in the clinical range. Despite the severe symptomatology reported by Fabio and the personality impairment observed by the therapist, the patient was situated in the non-clinical range in the initial assessment. Thus, statistically relevant changes were not expected for this patient. Second, qualitative data are not consistent with the hypothesis of a negative outcome. Indeed, Fabio reported several moderately to extremely important changes attributed to the therapy in the Change Interview. Furthermore, in the group supervision, the therapist reported important changes that defy standard evaluations. For example, we know that Fabio was overweight and lost weight during his therapy. Thus, an alternative hypothesis is that standard outcome measures are less suitable to capture therapeutic change in patients with psychic structure impairment.

Nevertheless, the deterioration observed in Fabio's CORE-OM scores requires reflection. Apparent deteriorations are expected in the early phases of some psychotherapy approaches. If the cognitive approach uses cognitive strategies to downregulate emotion, psychodynamic approaches—and more in general “expressive therapies”—are focused on affect recognition and expression (Greenberg and Pascual-Leone, 2006; Frederickson et al., 2018; Grecucci et al., 2018; Messina et al., 2020). As an example of expressive therapy, in ITAP sessions, the therapist is active in encouraging the patients' expression of their full experience of emotions and the associated impulses physically present in the body. This might be experienced as emotionally challenging by patients. Indeed, in previous studies, an initial trend to deterioration followed by a recovery toward positive outcomes has been noted as an effect of experiential and expressive techniques, such as imagery and chair work (van Asselt et al., 2008; Malogiannis et al., 2014). We consider that this temporary deterioration can be attributable to the progressive awareness of the patient's emotional difficulties in expressive therapies. For instance, it has been previously reported that some forms of deterioration in self-report questionnaires could reflect a less defensive attitude in the patients throughout therapy sessions (Mohr, 1995). In line with this interpretation, Fabio expressed the desire to continue his therapy after the end of this experience, suggesting an improved awareness concerning his psychological difficulties.

Finally, process examples reported in the present article may also help in reflecting the real efficacy of ITAP in the considered cases. As showed in the illustrative excerpts, despite the differences in available psychic resources in Maria's and Fabio's cases, both subjects were able to follow the therapist's analyses of intrapsychic and interpersonal triangles reaching the expression of their repressed impulses. The main difference between Maria and Fabio was that fewer psychic resources in Fabio required longer therapy and more caution in confrontations during the intervention, with the adoption of a supportive approach. In this regard, we consider that the observation of verbatim interactions of the therapeutic dyad is an irreplaceable element for the judgment of therapy effectiveness.

The results of the present study should be considered in light of the limitation of single-case methodology. Although patients involved in the study are representative of patients seen in clinical practice, any generalization of our results must be avoided due to the small number of patients considered. At the same time, exactly due to the specificity of single-case methodology, this study extended previous knowledge regarding the influence of ego strength on psychotherapy outcome by documenting the efficacy of ITAP therapy for patients with different ego strengths. Thus, we conclude that ego strength is not a limitation for the use of expressive therapy such as ITAP, but rather it is an important variable that should be considered to dose confrontations and support during psychotherapy sessions, with more support (and probably longer therapy) for patients with less ego strength.

## Data Availability Statement

The raw data supporting the conclusions of this article will be made available by the authors, without undue reservation.

## Ethics Statement

The studies involving human participants were reviewed and approved by Ethical Committee Psychology University of Padua (number 1703). The patients/participants provided their written informed consent to participate in this study. Written informed consent was obtained from the individual(s) for the publication of any potentially identifiable images or data included in this article.

## Author Contributions

IM: study planning, data analysis, and manuscript writing. AM: process material preparation. FS and MS: therapy provision, study planning, and ITAP conceptualization. EB: study planning and realization of change interview. AG: manuscript revision and supervision. All authors contributed to the article and approved the submitted version.

## Conflict of Interest

The authors declare that the research was conducted in the absence of any commercial or financial relationships that could be construed as a potential conflict of interest.

## References

[B1] Abbass A. (2015). Reaching Through Resistance: Advanced Psychotherapy Techniques. Kansas City: Seven Leaves Press.

[B2] American Psychiatric Association (2013). Diagnostic and statistical manual of mental disorders. 5th Edn. Washington, DC: Author. 10.1176/appi.books.9780890425596

[B3] Barkham M. Margison F. Leach C. Lucock M. Mellor-Clark J. Evans C.. (2001). Service profiling and outcomes benchmarking using the CORE-OM: toward practice-based evidence in the psychological therapies. J. Consult. Clin. Psychol. 69:184. 10.1037/0022-006X.69.2.184 11393596

[B4] Barron F. (1953). An ego-strength scale which predicts response to psychotherapy. J. Consult. Psychol. 17:327. 10.1037/h0061962 13109083

[B5] Berne E. (1961). Transactional Analysis in Psychotherapy. New York, NY: Grove Press.

[B6] Conte H. R. Plutchik R. Buck L. Picard S. Karasu T. B. (1991). Interrelations between ego functions and personality traits: Their relation to psychotherapy outcome. Am. J. Psychother. 45, 69–77. 10.1176/appi.psychotherapy.1991.45.1.69 2018198

[B7] CORE Partnership (2007). Is initial overall CORE-OM score an indicator of likely outcome? CORE Partnership Occasional Paper, No 1. CORE IMS: Rugby.

[B8] Davanloo H. (1994). Basic Principles and Techniques in Short-Term Dynamic Psychotherapy. New York, NY: Jason Aronson.

[B9] Duckworth A. L. Tsukayama E. May H. (2010). Establishing causality using longitudinal hierarchical linear modeling: an illustration predicting achievement from self-control. Soc. Psychol. Person. Sci. 1, 311–317. 10.1177/1948550609359707 20976121 PMC2957016

[B10] Elliott R. Slatick E. Urman M. (2001). “Qualitative change process research on psychotherapy: alternative strategies,” in Qualitative Psychotherapy Research: Methods and Methodology, eds J. Frommer and D. L. Rennie (Lengerich: Pabst Science), 69–111.

[B11] Evans C. Connell J. Barkham M. Margison F. McGrath G. Mellor-Clark J.. (2002). Towards a standardised brief outcome measure: psychometric properties and utility of the CORE—OM. Br. J. Psychiatry 180, 51–60. 10.1192/bjp.180.1.51 11772852

[B12] Fosha D. (2000). The Transforming Power of Affect: A Model of Accelerated Change. New York, NY: Basic Books.

[B13] Frederickson J. J. Messina I. Grecucci A. (2018). Dysregulated Anxiety and Dysregulating Defenses: toward an emotion regulation informed dynamic psychotherapy. Front. Psychol. 9:2054. 10.3389/fpsyg.2018.02054 30455650 PMC6230578

[B14] Freud S. (1923). The Ego and the Id. The Standard Edition of the Complete Psychological Works of Sigmund Freud. London: Hogarth Press.

[B15] Getter H. Sundland D. M. (1962). The Barron Ego Strength scale and psychotherapeutic outcome. J. Consult. Psychol. 26:195. 10.1037/h0047960 13898132

[B16] Goulding M. Goulding R. (1979). Changing Lives Through Redecision Therapy. California: Grove Press.

[B17] Grecucci A. Messina I. Amodeo L. Lapomarda G. Crescentini C. Dadomo H.. (2020a). A dual route model for regulating emotions: comparing models, techniques and biological mechanisms. Front. Psychol. 11:357. 10.3389/fpsyg.2020.00930 32581903 PMC7287186

[B18] Grecucci A. Messina I. Dadomo H. (2018). Decoupling internalized dysfunctional attachments: a combined ACT and schema therapy approach. Front. Psychol. 9:2332. 10.3389/fpsyg.2018.02332 30532729 PMC6265412

[B19] Grecucci A. Sigirci H. Lapomarda G. Amodeo L. Messina I. Frederickson J. (2020b). Anxiety regulation: from affective neuroscience to clinical practice. Brain Sci. 10:846. 10.3390/brainsci10110846 33198228 PMC7697078

[B20] Greenberg L. S. Pascual-Leone A. (2006). Emotion in psychotherapy: a practice-friendly research review. J. Clin. Psychol. 62, 611–630. 10.1002/jclp.20252 16523500

[B21] Hersoug A. G. Høglend P. Gabbard G. O. Lorentzen S. (2013). The combined predictive effect of patient characteristics and alliance on long-term dynamic and interpersonal functioning after dynamic psychotherapy. Clin. Psychol. Psychotherapy 20, 297–307. 10.1002/cpp.1770 22298434

[B22] Hill C. E. Knox S. Thompson B. J. Williams E. N. Hese S. Ladany N. (2005). Consensual qualitative research: An update. J. Couns. Psychol. 52, 196–205.

[B23] Laaksonen M. A. Knekt P. Sares-Jäske L. Lindfors O. (2013). Psychological predictors on the outcome of short-term psychodynamic psychotherapy and solution-focused therapy in the treatment of mood and anxiety disorder. Eur. Psychiatry 28, 117–124. 10.1016/j.eurpsy.2011.12.002 22705035

[B24] Lake B. (1985). Concept of ego strength in psychotherapy. Br. J. Psychiatry 147, 471–478. 10.1192/bjp.147.5.471 4075041

[B25] Lindfors O. Knekt P. Heinonen E. Virtala E. (2014). Self-concept and quality of object relations as predictors of outcome in short-and long-term psychotherapy. J. Affect. Disord. 152, 202–211. 10.1016/j.jad.2013.09.011 24091306

[B26] Malan D. H. (1976). The Frontier of Brief Psychotherapy. New York, NY: Plenum. 10.1007/978-1-4684-2220-7

[B27] Malogiannis I. A. Arntz A. Spyropoulou A. Tsartsara E. Aggeli A. Karveli S.. (2014). Schema therapy for patients with chronic depression: a single case series study. J. Behav. Ther. Exp. Psychiatry 45, 319–329. 10.1016/j.jbtep.2014.02.003 24650608

[B28] Menninger K. (1958). Theory of Psychoanalytic Technique. New York, NY: Basic Books. 10.1037/10843-000

[B29] Messina I. Grecucci A. Marogna C. Calvo V. (2020). Relational exposure and semantic processes as mechanisms of change in psychodynamic psychotherapy: convergences between psychotherapy research and affective neuroscience. Testing Psychometr. Methodol. Appl. Psychol. 27, 43–56. 10.4473/TPM27.1.3

[B30] Messina I. Scottà F. Benelli E. Bianco F. Sambin M. (2018). Intensive transactional analysis psychotherapy (ITAP): a single-case time series study. J. Psychol. Psychotherapy Res. 5, 46–52. 10.12974/2313-1047.2018.05.5

[B31] Messina I. Scottà F. Benelli E. Marchi A. Sambin M. (2019). Intensive transactional analysis psychotherapy (ITAP): a case series study. Counsell. Psychother. Res. 20, 222–232. 10.1002/capr.12277

[B32] Mohr D. C. (1995). Negative outcome in psychotherapy: a critical review. Clin. Psychol. 2, 1–27. 10.1111/j.1468-2850.1995.tb00022.x

[B33] Norcross J. C. Wampold B. E. (2011). What works for whom: tailoring psychotherapy to the person. J. Clin. Psychol. 67, 127–132. 10.1002/jclp.20764 21108312

[B34] OPD Task Force (2008). (ed.). Operationalized Psychodynamic Diagnosis OPD-2: Manual of Diagnosis and Treatment Planning. Cambridge MA: Hogrefe Publishing.

[B35] Palmieri G. Evans C. Hansen V. Brancaleoni G. Ferrari S. Porcelli P.. (2009). Validation of the Italian version of the clinical outcomes in routine evaluation outcome measure (CORE-OM). Clin. Psychol. Psychotherapy 16, 444–449. 10.1002/cpp.646 19701881

[B36] Rosenthal R. (1994). “Parametric measures of effect size,” in The Handbook of Research Synthesis, eds H. Cooper and L. V. Hedges (New York, NY: Russell Sage Foundation), 231–244.

[B37] Roth A. Fonagy P. (2006). What Works for Whom?: A Critical Review of Psychotherapy Research. New York, NY: Guilford Press.

[B38] Sambin M. (2018a). “ITAP: the theoretical model,” in Intensive Transactional Analysis Psychotherapy: An Integrated Model (ITAP), eds M. Sambin and F. Scottà (New York, NY: Routledge), 8.

[B39] Sambin M. (2018b). “Modulation of the intervention,” in Intensive Transactional Analysis Psychotherapy: An Integrated Model (ITAP), eds M. Sambin and F. Scottà (New York, NY: Routledge), 6.

[B40] Sambin M. Scottà F. (2018). Intensive Transactional Analysis Psychotherapy: An Integrated Model (ITAP). New York, NY: Routledge. 10.4324/9780203730850

[B41] Schiff J. L. (1975). The Cathexis Reader: Transactional Analysis Treatment of Psychosis. New York, NY: Harper and Row.

[B42] Scottà F. (2018). “Our idea of relational holding,” in Intensive Transactional Analysis Psychotherapy: An Integrated Model (ITAP), eds M. Sambin and F. Scottà (New York, NY: Routledge), 5.

[B43] Vaillant G. E. (1992). Ego *M*echanisms of *Defense: A Guide for Clinicans and Researchers*. Washington, DC: American Psychiatric Pub.

[B44] van Asselt A. D. Dirksen C. D. Arntz A. Giesen-Bloo J. H. van Dyck R. Spinhoven P.. (2008). Out-patient psychotherapy for borderline personality disorder: cost-effectiveness of schema-focused therapy V. Transference focused psychotherapy. Br. J. Psychiatry 192, 450–457. 10.1192/bjp.bp.106.033597 18515897

[B45] Vittengl J. R. Clark L. A. Thase M. E. Jarrett R. B. (2016). Defined symptom-change trajectories during acute-phase cognitive therapy for depression predict better longitudinal outcomes. Behav. Res. Ther. 87, 48–57. 10.1016/j.brat.2016.08.008 27591917 PMC5127736

